# Correlation Between Blood Lipid Levels and Chronic Pancreatitis: A Retrospective Case–Control Study of 48 Cases

**DOI:** 10.1097/MD.0000000000000331

**Published:** 2014-12-02

**Authors:** Qingqiang Ni, Lin Yun, Rui Xu, Dong Shang

**Affiliations:** From the Medical College of Soochow University (QN), Suzhou, Jiangsu; Eastern Hepatobiliary Surgery Hospital (QN), Shanghai; Department of General Surgery (QN, DS), Pancreato-Biliary Center, First Affiliated Hospital, Dalian Medical University, Dalian, Liaoning; Jinan Maternity and Child Care Hospital (LY), Jinan, Shandong; and Department of Cardiology (RX), Shandong Provincial Qianfoshan Hospital, Shandong University, Jinan, Shandong, P.R. China.

## Abstract

The incidence of chronic pancreatitis (CP) is increasing, and dyslipidemia severely affects the health of middle-aged and elderly people. We investigated the association between blood lipid levels and CP.

The serum lipid metabolic indices of 48 patients with CP (CP group) were summarized retrospectively. The physical examination results of 40 randomly selected healthy individuals were used as the normal control (NC) group. Statistical analyses of the blood lipid data were performed between the 2 groups using the case–control study method.

High-density lipoprotein-cholesterol (HDL-c) levels decreased and fasting blood glucose (GLU) levels increased in the CP group compared with those in the NC group (*P* < 0.01). Pearson correlation analysis results showed that serum amylase (AMY) was positively correlated with low-density lipoprotein-cholesterol (LDL-c; *r* = 0.414, *P* < 0.05), and urine AMY (UAMY) was positively correlated with total cholesterol (TC; *r* = 0.614, *P* < 0.01) and LDL-c (*r* = 0.678, *P* < 0.01). A binary logistic regression analysis showed that GLU (odds ratio [OR], 5.052; *P* < 0.01) and TC (OR, 1.074; *P* < 0.01) may be risk factors for CP, whereas HDL-c may be a CP protective factor (OR, 0.833; *P* < 0.01).

The HDL-c levels decreased and GLU levels increased in the CP group compared with those in the NC group; AMY was positively correlated with LDL-c and UAMY was positively correlated with TC and LDL-c; GLU and TC may be risk factors for CP; and HDL-c may be a CP protective factor. This may be the first time that such results have been reported. These findings will contribute to primary prevention and control of CP progression.

## INTRODUCTION

Chronic pancreatitis (CP) refers to limiting, segmental, diffusing, progressive inflammatory damage, necrosis, and interstitial fibrous lesion of the pancreatic parenchyma because of many causes, usually accompanied by stenosis and dilation of the pancreatic duct, pancreatic calcification, and pancreatic stone formation. The necrosis of pancreatic acinar cells, the atrophy or loss of pancreatic islet cells, and extensive interstitial fibrosis will eventually result in the irreversible destruction of the pancreatic morphology and structure as well as exocrine and endocrine pancreatic insufficiency.

The incidence of CP has geographical differences. According to Liao et al, the incidence of CP is approximately 42/100,000 in USA, 26/100,000 in France, and 22/100,000 in Japan; the incidence was the highest in India at approximately 114 to 200/100,000.^1–4^ The results of a survey performed in 2008 showed that during 10 years between 1994 and 2004, the incidence of CP in 22 hospitals in China was approximately 13/100,000 and showed an increasing trend year by year.^5^ It was reported that the ages of 50% of patients with CP were 40 to 59 years; patients >60 years of age accounted for 15%; patients <40 accounted for 35%; 79% of patients were males, while 21% were females; the incidence of CP in black people was higher than in white people; and the 5-year survival rate was >95%.^6^

The etiology of CP is multifactorial, the result of joint action by environmental and genetic factors.^7^ In North America, CP caused by alcohol abuse accounts for 44.5%, nonalcoholic CP for 26.9%, and idiopathic CP for 28.6% of cases. Alcohol abuse is the major cause of CP in males, and the ratio of males to females is 59.4%/28.1%; in nonalcohol abuse condition, the ratio of males to females is 18%/36.7%. Idiopathic causes are more common in females, and the male-to-female ratio is 22.6%/35.2%, *P* < 0.01.^8^ In western countries, excessive alcohol consumption plays a leading role and accounts for 60% of cases of CP. In China, excess alcohol consumption only accounts for 35% of the cases of CP.^2^

In China, as quality of life continues to increase, excess alcohol consumption has gradually replaced biliary tract disease as the leading cause of CP in China. According to a multicenter survey on CP in China, CP caused by biliary tract disease accounted for 33.9%, CP caused by long-term excess alcohol consumption for 35.4%, and CP caused by pancreatic trauma for 10.5% of cases.^9^ The major causes of biliary tract disease include cholelithiasis, choledocholithiasis, cholecystitis, dysfunction of the papillary muscle (spasm or stricture), and biliary ascariasis. The damage to exocrine pancreatic acinar cells and endocrine islet cells caused by the reflux of bile into the pancreatic duct and fibrosis caused by interstitial fibrous connective tissue proliferation are the major etiologies. A comparison between smokers and nonsmokers showed that the age of onset in smokers was earlier than in nonsmokers, and the incidence of pancreatic calcification and diabetes mellitus was higher in smokers than in nonsmokers.^8^ Other reasons such as gene mutation or loss, hypercalcemia, autoimmune diseases, hyperlipidemia, and congenital malformation of the pancreas can also cause CP.^10^ However, no studies on the correlation between dyslipidemia and CP have been reported. Whether dyslipidemia is correlated with the development of CP is worthy of further investigation.

Exocrine pancreatic acinar cell damage occurs in the early stage of CP lesions. With disease progression, endocrine islet cells are involved in the late stage, and a large amount of progressive interstitial fibrous tissue proliferation replaces the normal pancreatic parenchyma cells. Based on the histopathological changes to the pancreas, CP can be classified into 3 types.

1. Chronic obstructive pancreatitis: Degeneration, necrosis, and infection of pancreatic parenchyma cells invade the pancreatic duct, thus causing stenosis of the pancreatic duct. Protein plugs are rarely found in the inside tube; the pancreatic duct epithelium is well preserved, there is no stenosis inside the tube, and there is no pancreatic duct calcification.
2. Chronic calcifying pancreatitis: This is the most common type of CP and includes alcoholic CP. The lesion shows a punctate shape. Some dilated branches of the pancreatic duct have irregular morphology. The main pancreatic duct exhibits stenosis, dilation, calcification, or even stones. The epithelial atrophy of the pancreatic duct and ductal protein embolism often occur.
3. Chronic inflammatory pancreatitis: CP resulting from chronic inflammation caused by chronic inflammation of the biliary tract and stenosis induced by scar formation belongs to this type. The presentations are the destruction of pancreatic exocrine parenchymal cells, formation of diffuse fibrosis, and mononuclear cell infiltration.^11^

With the prominence of the problems of population aging, the incidence of CP shows an increasing trend year by year; dyslipidemia also severely affects the health condition of middle-aged and aged people. The relationship between these two warrants attention. Therefore, this study involved case–control studies on the association between blood lipid levels and CP to provide reference bases for the future diagnosis and treatment of CP.

## POPULATION AND METHODS

### Population

#### General Information

This case–control study was approved by the Ethics Committee of the First Affiliated Hospital of Dalian Medical University (Dalian, Liaoning, China). Written informed consents were obtained from the patient for publication of this case–control study. A retrospective analysis was performed on the measurement results of 4 blood lipid indicators in 48 patients with CP (CP group) treated in the First Affiliated Hospital of Dalian Medical University between March 2011 and March 2014. In addition, the blood lipid data of 40 healthy individuals who received physical examination in the First Affiliated Hospital of Dalian Medical University between March 2011 and March 2014 were used as the normal control (NC) group. The gender, age, smoking, drinking, and body mass index (BMI) conditions of the NC group were comparable to those of the CP group. Pack-years was calculated as years of smoking multiplied by the average number of packages smoked per day. A package contains 20 cigarettes. The drinking index was calculated as years of drinking multiplied by the average grams of alcohol consumed per day. Subjects were defined as cigarette smokers or alcohol drinkers if they had smoked ≥5pack-years or had a ≥150 drinking index, respectively. Nineteen of the 48 CP cases were diagnosed with diabetes, and 10 of the 19 cases were administered metformin. Five of the 19 cases were treated with insulin, and 4 of the 19 cases had already been diagnosed with diabetes and were not treated. GLU levels in 10 of the 15 treated cases were controlled to normal levels, whereas 5 cases were poorly controlled.

#### Inclusion and Exclusion Criteria

The inclusion criteria for cases in the CP group were primarily based on the guidelines for the diagnosis and treatment of CP released by the Chinese Society of Digestive Endoscopy in Shanghai in 2012: (1) typical clinical manifestations (eg, recurrent upper abdominal pain, acute pancreatitis); (2) pancreatic calcification, pancreatic duct stones, and pancreatic duct stenosis or dilatation suggested by imaging examination; (3) characteristic changes in pathology; and (4) presentation of exocrine pancreatic insufficiency. Patients with (2) or (3) were confirmed cases, and patients with (1) and (4) were probable cases. The following imaging methods were used to diagnose CP. (1) Endoscopic ultrasonography was used to detect pancreatic stones, dilation of the side branches, stenosis or irregular dilation of the main pancreatic duct, increased pancreatic parenchymal intensity by ultrasound, and pseudocysts. (2) Magnetic resonance imaging/magnetic resonance cholangiopancreatography was used to detect irregular dilation of the pancreatic ducts, focal pancreatic enlargement, parenchymal atrophy, dilation of the biliary duct, peripancreatic fluid, irregular contour of the pancreas, and disruption of the pancreatic duct. (3) Computed tomography was used for pancreatic calcification, irregular dilation of the pancreatic ducts, focal pancreatic enlargement, parenchymal atrophy, dilation of the biliary duct, peripancreatic fluid, irregular borderline or shape of the pancreas, and pseudocysts. (4) Endoscopic retrograde cholangiopancreatography was used to assess dilation or/and obstruction of side branches, dilation, stenosis, obstruction, calcification and/or stones in the main pancreatic duct, and pseudocysts. The exclusion criteria for cases in the CP group were as follows: patients with insufficient clinical information or without blood lipid indicator measurement.

The inclusion criteria of the NC group were healthy individuals with age, gender, smoking, drinking, and BMI conditions similar to the CP group to minimize the influence of age, gender, smoking, drinking, and BMI on the statistical results. The exclusion criteria of cases in the NC group were as follows: people with bad habits; and people with biliary diseases or a history of biliary diseases shown by physical examination reports.

### Methods

#### Contents of Retrospective Analysis of Cases

GLU and the 4 blood lipid indicators included total cholesterol (TC), triglyceride (TG), low-density lipoprotein-cholesterol (LDL-c), and high-density lipoprotein-cholesterol (HDL-c). The determination criteria were as follows: normal range TC < 200 mg/dL, TG 50 to 150 mg/dL, LDL-c < 120 mg/dL, HDL-c > 40 mg/dL, and GLU 3.9 to 6.1 mmol/L.

#### Statistical Analysis

Statistical analysis was performed using the SPSS17.0 software (SPSS Inc, Chicago, IL). The measurement data were presented as‘x¯ ± s. Comparison between the 2 groups was performed using the 2 independent samples *t* test. Count data were examined using the χ^2^ test. Correlation analysis was performed using Pearson correlation analysis. The analysis of risk factors was performed using the binary logistic regression. *P* < 0.05 indicated that the difference was statistically significant.

## RESULTS

### Comparison of Gender, Age, Smoking, Drinking, and BMI Conditions Between the CP and NC Groups

There were 48 cases in the CP group, 27 males and 21 females; the mean age was (60.85 ± 13.36) years; there were 18 smokers and 16 drinkers; and the mean BMI was (25.48 ± 4.75) kg/m^2^. There were 40 cases in the NC group, 20 males and 20 females; the mean age was (56.30 ± 10.92) years; there were 11 smokers and 8 drinkers; and the mean BMI was (26.18 ± 4.98) kg/m^2^. The differences in gender, age, smoking, drinking, and BMI between these 2 groups all lacked statistical significance (*P* > 0.05) and had comparability (Table 1).

**TABLE 1:**
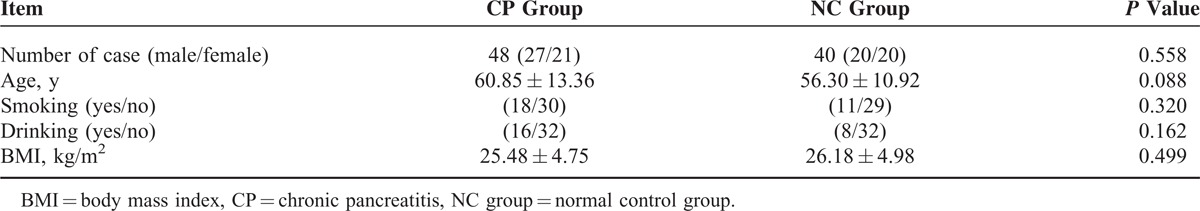
Comparison of Gender, Age, Smoking, Drinking, and BMI Between the CP and NC Groups

### Comparison of GLU and Blood Lipid Indicators Between the CP and NC Groups

The TC, TG, and LDL-c levels exhibited no significant difference between the CP and NC groups (*P* > 0.05), while the levels of HDL-c in the CP group were significantly lower than in the NC group (*P* < 0.01) and the levels of GLU in the CP group were significantly higher than those in the NC group (*P* < 0.01) (Table 2).

**TABLE 2:**
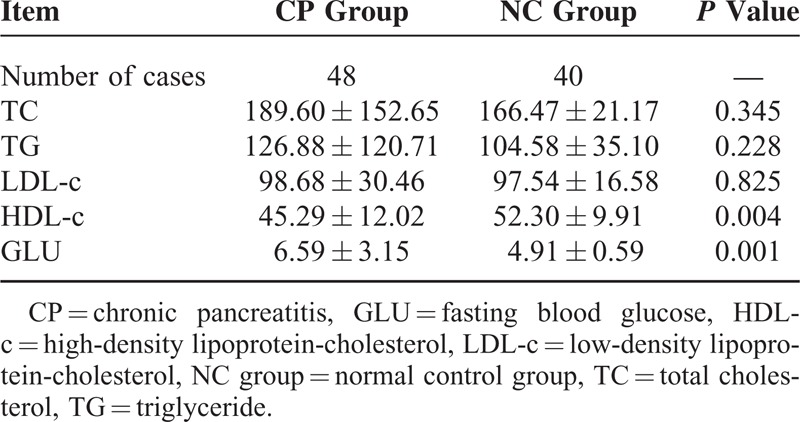
Comparison of TC, TG, LDL-c, HDL-c, and GLU Between the CP and NC Groups (x¯ ± s)

### Pearson Correlation Analysis

Serum amylase (AMY) did not correlate with the TC, TG, and HDL-c levels (*P* > 0.05) but did exhibit a positive correlation with the LDL-c levels (*r* = 0.414, *P* < 0.05). Urine amylase (UAMY) did not correlate with the TG and HDL-c levels (*P* > 0.05) but did exhibit a positive correlation with the TC levels (*r* = 0.614, *P* < 0.01) and with the LDL-c levels (*r* = 0.678, *P* < 0.01). Lipase did not correlate with the TC, TG, LDL-c, and HDL-c levels (*P* > 0.05) (Table 3).

**TABLE 3:**
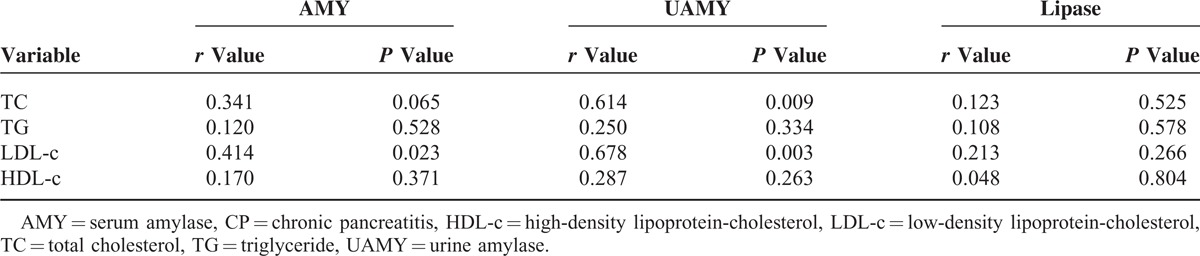
Pearson Correlation Analyses Between AMY, UAMY, Lipase, and Blood Lipid of Patients in the CP Group

### Binary Logistic Regression Analysis

Results of binary logistic regression analysis on the correlations between all indicators and the risk of CP showed that after gender, age, smoking, drinking, and BMI were adjusted, GLU and TC may be risk factors for CP and *P* value is 0.003 and 0.009, respectively (*P* < 0.01) and odds ratio (OR) value is 5.052 and 1.074, respectively; and HDL-c may be a CP protective factor (OR, 0.833; *P* < 0.01) (Table 4)

**TABLE 4:**
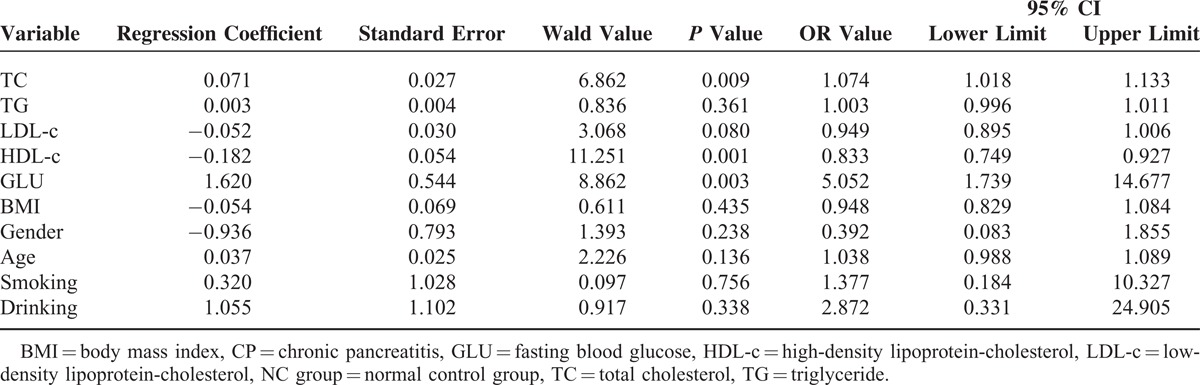
Binary Logistic Regression Analysis on the Correlation Between All Indicators and the Risk of CP

## DISCUSSION

With the increasing prominence of the problems of population aging, the incidence and mortality rates of cardiovascular and cerebrovascular diseases caused by atherosclerosis have exhibited an increasing trend year by year. “Blood lipids” is the general term for various lipids in the blood. The level of blood lipids in the body of healthy individuals is maintained at a dynamic balance; the detection of blood lipids can directly reflect the metabolic condition of lipids inside the body. An abnormal lipid metabolism plays an important role in the formation of atherosclerotic plaques.^12^ A large number of studies have confirmed that TC, TG, and LDL-c are major risk factors for cardiovascular diseases, whereas HDL-c has a negative correlation with cardiovascular diseases.^13–15^ In clinical contexts, the routine detection of blood lipid indicators has received increasing attention from medical personnel in all departments.

It has been reported that the risk factors for CP development include drinking, smoking, diabetes mellitus, gene mutations of calcium-sensitive receptors, volatile hydrocarbons in occupation, celiac disease, aspirin abuse, chronic renal failure, toxins, recurrent acute pancreatitis, severe acute pancreatitis, pancreatic duct obstruction, hypercalcemia, and hyperlipidemia.^6,7,16–18^ The pathogenesis of CP is still not clear. For patients without high risk factors, the current consensus is that alcoholism, biliary pancreatitis, gene mutations [serine protease inhibitor, *CFTR* (cystic fibrosis transmembrane conductance regulator) gene; protease, serine, 1 (trypsin 1); and serine peptidase inhibitor, Kazal type 1], idiopathic pancreatitis, irregular lifestyles, and autoimmune diseases may be the causes of CP.^19^

A clinical study by Zhang et al^20^ showed that compared with healthy individuals, patients with CP had elevated plasma levels of glucose, lactate, creatine, formic acid, glycerol, tyrosine, phenylalanine, lysine, histidine, glutamine, glutamic acid, and alanine; in addition, patients with CP also exhibited decreased LDL, very low-density lipoprotein, and 3-hydroxyacetone levels. However, there was no significant difference in the HDL-c levels between the healthy control and the CP group.^20^ In our study, all the biochemical indicators were obtained in the Department of Laboratory Medicine of our hospital using the same measurement procedures and conditions. The NC group included healthy individuals who received physical examination in our hospital; their age, gender, smoking, drinking, and BMI conditions were not significantly different from the CP group and had comparability. The study results showed that the HDL-c level in the CP group was significantly lower than in the NC group (*P* < 0.01). The TC, TG, and LDL-c levels between the CP and NC groups did not exhibit any significant difference (*P* > 0.05). Chronic inflammation can reduce HDL-c levels.^21^ Lecithin/cholesterol acyltransferase, platelet-activating factor-acetyl hydrolase, and paraoxonase 1 are HDL-associated enzymes. Under inflammatory conditions, they can become dysfunctional or/and depleted.^22,23^ These might be the reasons why HDL-c level was decreased in the CP group. Our results were somewhat different from the results of Zhang et al,^20^ which might be attributable to the different study methods and small included sample size of the 2 studies. It remains necessary for researchers to conduct additional multicenter and large sample cohort studies for verification and supplementation.

In the early stage of CP, acute attack may be accompanied by increased AMY and UAMY; however, in the late stage, AMY and UAMY are usually not increased, or the increase is not significant. Lipase levels are usually not significantly increased in patients with CP. In fact, except for a slight increase during acute attack, lipase levels usually show different degrees of decrease in other situations. In light of limited patient data, this study performed Pearson correlation analyses between the AMY, UAMY, and lipase levels and the TC, TG, LDL-c, and HDL-c levels. The results showed that the AMY and LDL-c levels exhibited a positive correlation (*r* = 0.414, *P* < 0.05), while UAMY was positively correlated with the TC and LDL-c levels (*r* = 0.614, *r* = 0.678, *P* < 0.01). Low-density lipoprotein particles are the main carriers of cholesterol into the arterial wall. Endothelial dysfunction can be caused by chronic exposure to elevated LDL-c levels in the blood, resulting in chronic inflammation.^24^ The chronic release of inflammatory mediators may cause direct damage to pancreatic acinar cells, resulting in the release of pancreatic amylase into the blood. These might be the reasons why LDL-c showed significant correlation with AMY and UAMY. These results indicated that the increased AMY and UAMY levels in patients with CP might be accompanied by increased TC and LDL-c levels. The clinical study of Yadav et al^25^ on patients with diabetes mellitus showed that these patients with low serum HDL-c levels were usually accompanied by low levels of AMY.^25^ The clinical study by Yingjie et al^26^ on acute hyperlipidemic pancreatitis confirmed that serum TG levels did not correlate with AMY levels in patients with acute hyperlipidemic pancreatitis. Our studies showed that the serum TG levels and AMY levels did not exhibit any correlation in patients with CP. This result might be because acute hyperlipidemic pancreatitis and CP shared the common etiological factor of hyperlipidemia. The mechanism underlying the induction of acute pancreatitis and CP by hyperlipidemia is currently not clear. Pancreatic lipase can hydrolyze blood lipids into free fatty acids, which might be the result of direct damage to pancreatic acinar cells.

At least 90% of the normal gland is dysfunctional when steatorrhea, diarrhea, and other malabsorption symptoms develop. Only 2 cases of steatorrhea were detected in the 48 cases of CP, and it may have been relevant to select patients without such severe CP. Thus, lipid profile data of 46 of the 48 patients in the CP group and the 40 control group cases were statistically analyzed. The results assessed with and without the steatorrhea cases using 2 independent sample *t* tests showed that the HDL-c levels in the CP group were significantly lower than those in the NC group (*P* = 0.004 and 0.006, respectively). The GLU levels in the CP group were significantly higher than those in the NC group (*P* = 0.001). Both results with and without steatorrhea patients using binary logistic regression analysis showed *P* values of 0.001 and the OR values for HDL-c were 0.833 and 0.838, respectively. The *P* values were 0.003 and 0.005, respectively, and the OR values for GLU were 5.052 and 4.604, respectively. The *P* values with and without steatorrhea cases were 0.009 and 0.008, respectively, and the OR values for TC were 1.074 and 1.076, respectively. These data suggest that GLU and TC may be the risk factors for CP and that HDL-c may be a protective factor for CP, regardless of whether the 2 steatorrhea cases were included. It has been reported that steatorrhea develops when lipase output is <10% than that of normal output.^27^ Quantifying fecal fat is the gold standard for diagnosing pancreatic exocrine insufficiency.^28^ However, this method requires a strict diet of 100 g fat/d for 5 days, and feces from the last 3 days must be collected. Thus, a major drawback is that the process is cumbersome and unpleasant for both laboratory personnel and patients. Therefore, this method is only available at a few specialized centers. Moreover, quantifying fecal fat is a common way to evaluate fat malabsorption in patients with CP. However, almost no data have been reported on the correlation between steatorrhea and HDL-c.

To the best of our knowledge, no previous domestic or international report had shown similar conclusions. Therefore, we speculated that the increased HDL-c levels and decreased GLU or/and TC levels may exert a certain preventive and protective effect on the development of CP. Before application of this conclusion in clinical practice, we suggest that researchers conduct further multicenter and large sample cohort studies for verification.

## CONCLUSION

The HDL-c levels decreased and GLU levels increased in the CP group compared with those in the NC group; AMY was positively correlated with LDL-c and UAMY was positively correlated with TC and LDL-c; GLU and TC may be risk factors for CP; and HDL-c may be a CP protective factor. This may be the first time that such results have been reported. These findings will contribute to primary prevention and control of CP progression.
